# Supplementary material for: Selective targeting of ligand-dependent and -independent signaling by GPCR conformation-specific anti-US28 intrabodies
Source: Nat Commun. 2021 Jul 16;12:4357. doi: 10.1038/s41467-021-24574-y (PMC8285524; doi:10.1038/s41467-021-24574-y)
Supplement: Supplementary file 1 — Supplementary Information [file 41467_2021_24574_MOESM1_ESM.pdf]

Supplementary Materials

Selective targeting of ligand-dependent and -independent signaling by GPCR conformation-specific anti-US28 intrabodies

Authors:

Timo W.M. De Groof, Nick D. Bergkamp, Raimond Heukers, Truc Giap, Maarten P. Bebelman, Richard Goeij-de Haas, Sander R. Piersma, Connie R. Jimenez, K. Christopher Garcia, Hidde L. Ploegh, Marco H. Siderius and Martine J. Smit

---

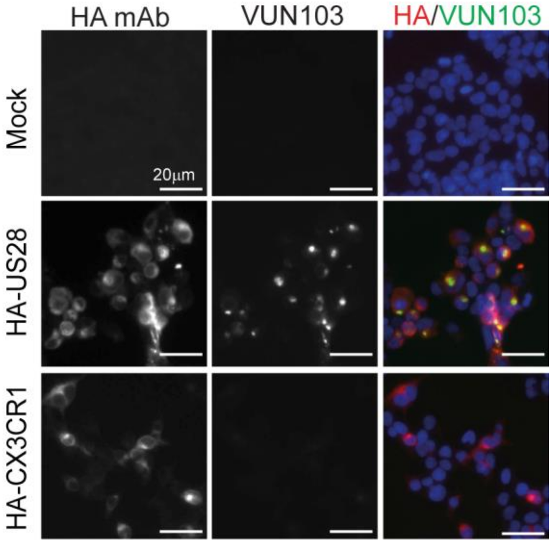

Supplementary Figure 1. VUN103 binds selectively to US28. Immunofluorescence microscopy of the binding of VUN103 to US28 and CX3CR1 receptor. HEK293T cells were transiently transfected with empty vector (Mock) or with DNA encoding HA-tagged US28 (HA-US28) or HA-tagged CX3CR1 (HA-CX3CR1) receptor. Cells were fixed and permeabilized before receptor expression was detected using an N-terminal HA-tag and anti-HA antibody (HA mAb; HA, red). VUN103 binding was detected using the Myc-tag and an anti-Myc antibody (VUN103, green). Representative data of 3 independent experiments.

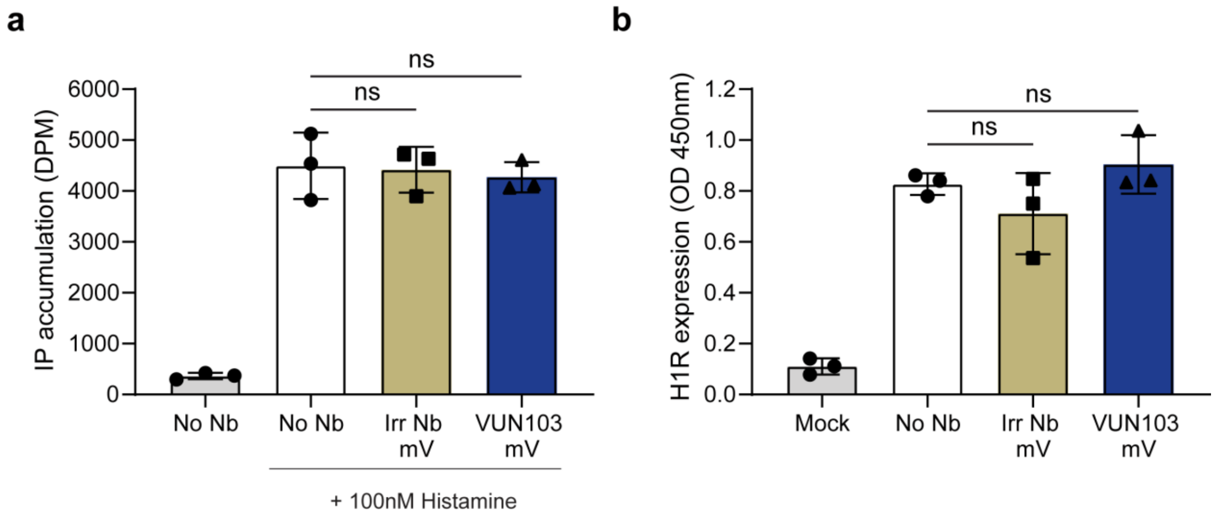

Supplementary Figure 2. Effect of VUN103-mVenus on histamine H1 receptor activation. a) Effect of non-US28 targeting intrabody-mVenus (Irr Nb mV) or VUN103-mVenus (VUN103 mV) on Histamine 1 Receptor-mediated accumulation of inositol phosphate (IP) upon stimulation with histamine (100 nM). HEK293T cells were transfected with DNA encoding HA-tagged Histamine 1 receptor (No Nb) or HA-tagged Histamine 1 receptor and Irr Nb mV or VUN103 mV (n=3 independent experiments). **b)** Determination of Histamine 1 receptor (H1R) expression using ELISA on HEK293T cells transfected with empty vector (Mock), DNA encoding HA-tagged Histamine 1 receptor (No Nb) or HA-tagged Histamine 1 receptor and Irr Nb mV or VUN103 mV. Receptor expression was detected using the N-terminal HA-tag and an anti-HA antibody (n=3 independent experiments). All data is plotted as mean ± S.D. Statistical analyses were performed using unpaired two-tailed t-test. ns, $p > 0.05$. Source data are provided as a Source Data file.

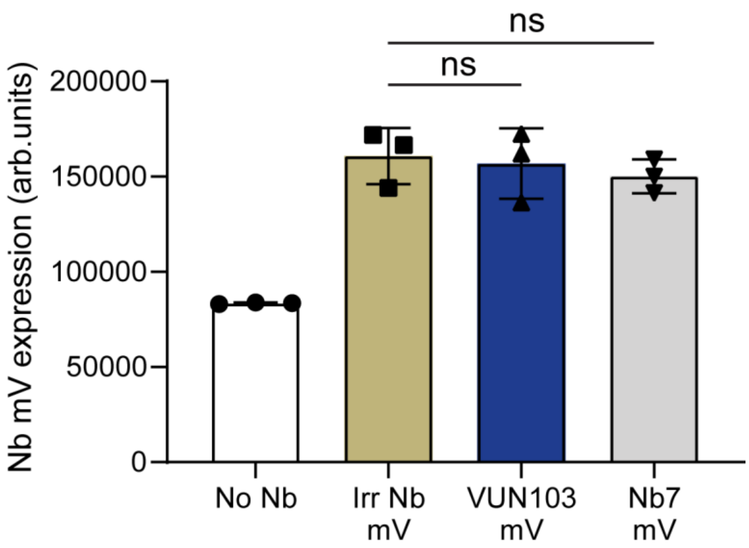

Supplementary Figure 3. Determination of intrabody-mVenus expression levels. HEK293T cells were transfected with DNA encoding HA-US28 (No Nb) or HA-US28 and non-US28 targeting intrabody-mVenus (Irr Nb mV), VUN103-mVenus (VUN103 mV) or Nb7-mVenus (Nb7 mV). Intrabody-mVenus (Nb mV) expression levels were determined by measuring mVenus fluorescence intensity (n=3 independent experiments). Data is plotted as mean ± S.D. Statistical analyses were performed using unpaired two-tailed t-test. ns, $p > 0.05$. Source data are provided as a Source Data file.

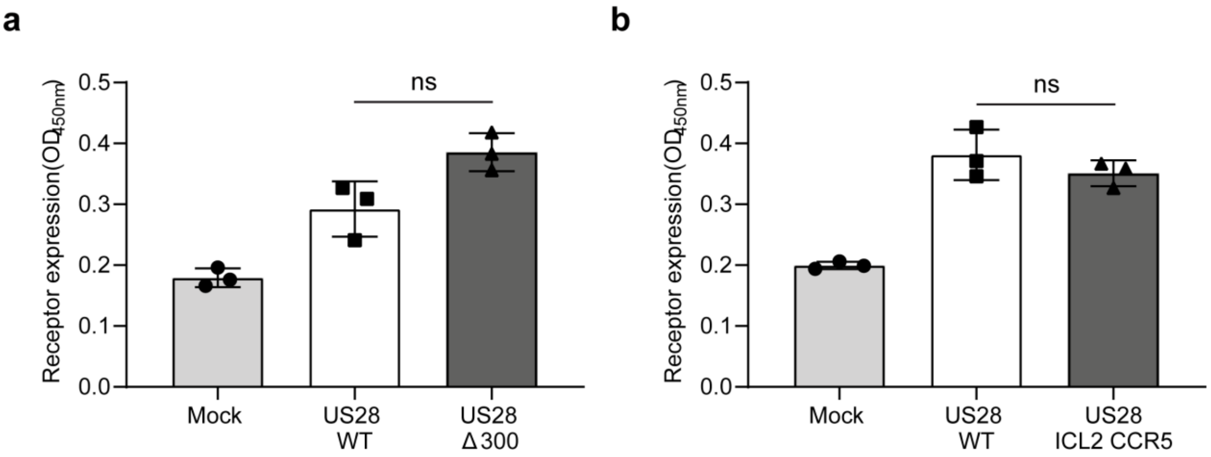

Supplementary Figure 4. Receptor expression of US28-transfected HEK293T cells. ELISA on HEK293T cells transfected with empty vector (Mock), vector encoding HA-US28 wildtype receptor (US28 WT), vector encoding HA-US28 lacking a C-tail (Δ300, **a**) or an HA-US28 chimera with the ICL2 loop substituted with the corresponding ICL2 loop of CCR5 (US28 ICL2 CCR5, **b**). Receptor expression was determined by the N-terminal HA-tag and an anti-HA antibody (n=3 independent experiments). Data is plotted as mean ± S.D. Statistical analyses were performed using unpaired two-tailed t-test. ns, $p > 0.05$. Source data are provided as a Source Data file.

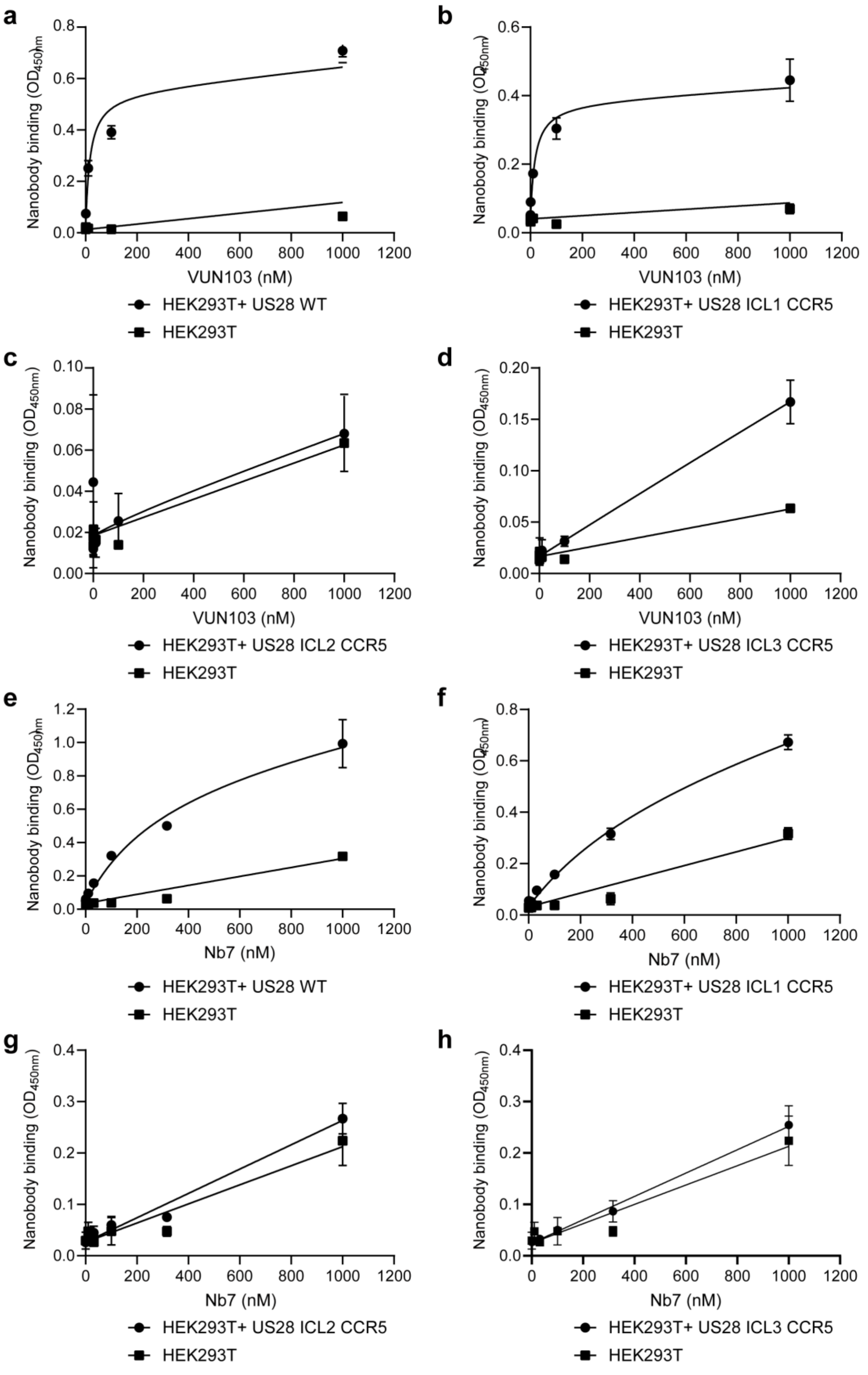

Supplementary Figure 5. Affinity determination of VUN103 and Nb7 to different US28 ICL mutants. Binding of different concentrations of the nanobody VUN103 and Nb7 to control HEK293T

(HEK293T) membranes and HEK293T membranes expressing US28 wildtype (HEK293T+ US28 WT, **a-e**), US28-ICL1-CCR5 chimera (HEK293T+ US28 ICL1 CCR5, **b-f**), US28-ICL2-CCR5 chimera (HEK293T+ US28 ICL2 CCR5, **c-g**) or US28-ICL3-CCR5 chimera (HEK293T+ US28 ICL3 CCR5, **d-h**) was determined by ELISA (n=3 independent experiments). All data is plotted as mean ± S.D. Source data are provided as a Source Data file.

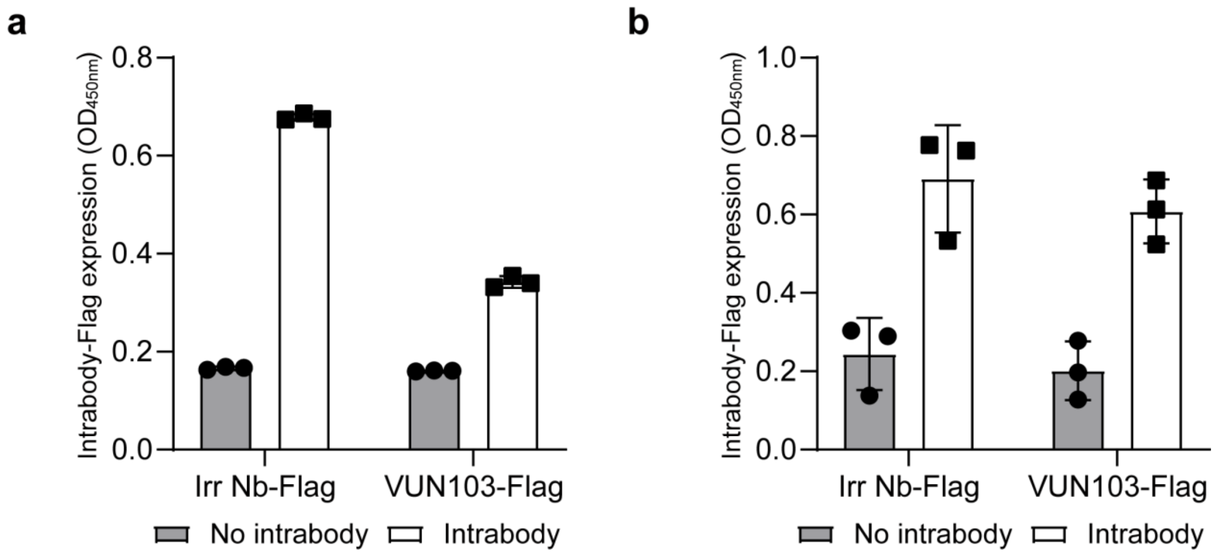

Supplementary Figure 6. FLAG-Tagged intrabody expression of US28-NLuc transfected HEK293T cells. ELISA on HEK293T cells transfected with vector encoding US28-NLuc receptor, mVenus-mini- Gα_q (**a**) or ß-arrestin2-mVenus (**b**) and inducible FLAG-tagged non-US28 targeting intrabody (Irr Nb-FLAG) or inducible FLAG-tagged VUN103 (VUN103-FLAG). Expression of FLAG-tagged intrabodies was not induced (No intrabody) or induced using 100 nM of tebufenozide, two days prior to the BRET read-out (Intrabody). FLAG-tagged intrabody expression was determined by the C-terminal FLAG-tag and an anti-FLAG antibody (n=3 independent experiments). All data is plotted as mean ± S.D. Source data are provided as a Source Data file.

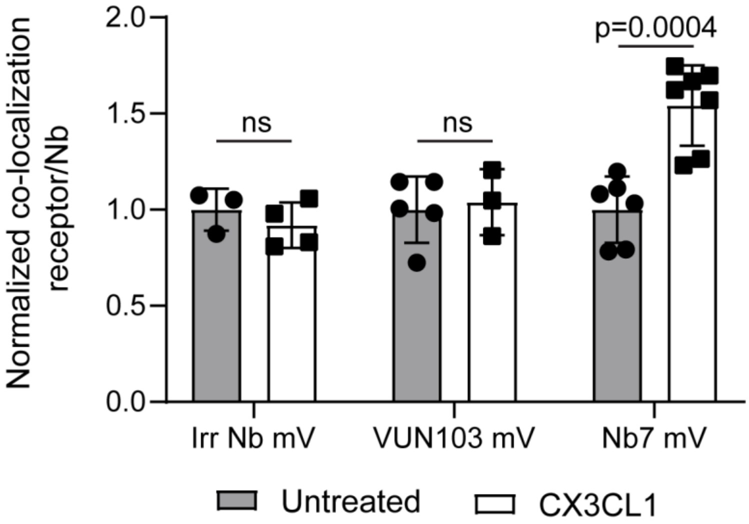

Supplementary Figure 7. Quantification of co-localization of US28 and nanobody-mVenus upon fractalkine binding. Co-localization of (surface) US28 and non-US28 targeting intrabody-mVenus (Irr Nb mV; n=3 untreated cells and n=4 CX3CL1-treated cells), VUN103-mVenus (VUN103 mV n=4 untreated cells and n=3 CX3CL1-treated cells) or Nb7-Venus (Nb7 mV n=6 untreated cells and n=7 CX3CL1-treated cells) 20 min after no treatment (Untreated) or addition of 30 nM of CX3CL1 (CX3CL1). Co-localization was quantified using ImageJ and JACoP plugin. Co-localization signal was normalized to the signal of untreated HEK293T cells expressing HA-US28 and nanobody-mVenus (Nb). Experiment was performed twice and data is plotted as mean ± S.D. Statistical analyses were performed using unpaired two-tailed t-test. ns, $p > 0.05$. Source data are provided as a Source Data file.

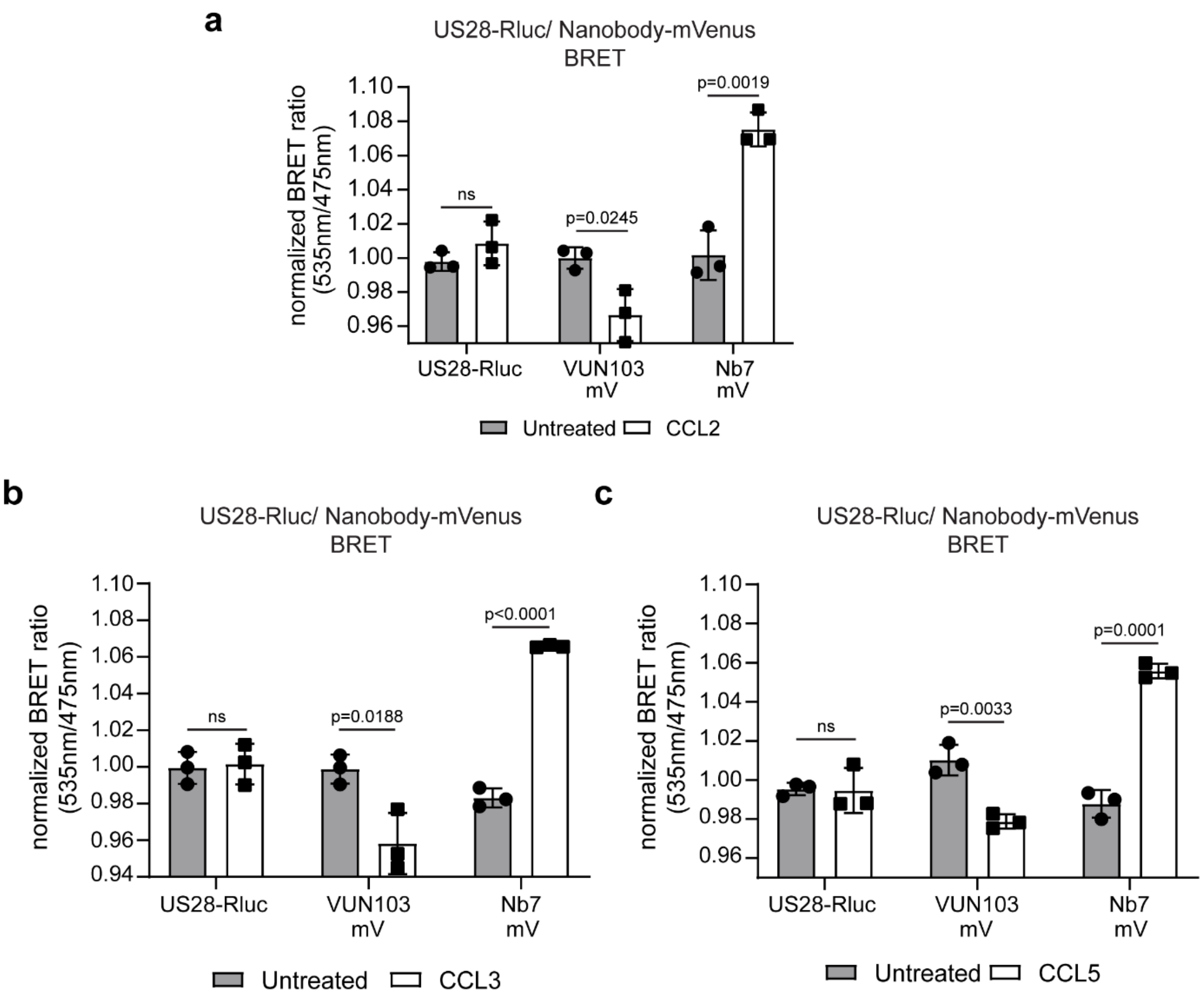

Supplementary Figure 8. BRET of US28-Rluc and nanobody-mVenus upon ligand binding. a-c) BRET using HEK293T cells expressing US28-Renilla luciferase (US28-Rluc), US28-Rluc and VUN103-mVenus (VUN103 mV) or Nb7-mVenus (Nb7 mV) 20 min after no treatment (Untreated) or addition of 100 nM CCL2 (**a**), CCL3(**b**) or CCL5 (**c**). BRET signal was normalized to the signal of unstimulated HEK293T cells expressing US28-Rluc only at timepoint t=0 (n=3 independent experiments). All data is plotted as mean ± S.D. Statistical analyses were performed using unpaired two-tailed t-test. ns, $p > 0.05$. Source data are provided as a Source Data file.

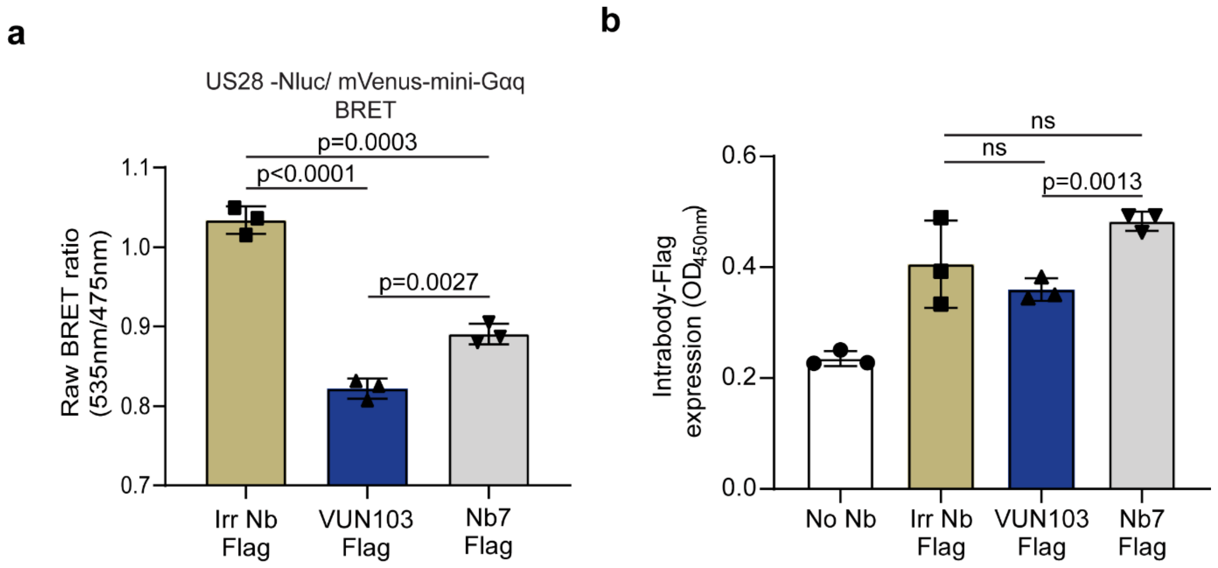

Supplementary Figure 9. Effect of intrabody-FLAG expression on BRET of US28-Nluc and mVenus-mini-Gα_q a) Raw BRET with HEK293T cells expressing US28-Nluc, mVenus-mini-Gα_q and an inducible FLAG-tagged non-US28 targeting intrabody (Irr Nb-FLAG), FLAG-tagged VUN103 (VUN103-FLAG) or FLAG-tagged Nb7 (Nb7-FLAG). Expression of FLAG-tagged intrabodies was induced using 100 nM of tebufenozide, one day prior to read-out (n=3 independent experiments). **b)** ELISA on HEK293T cells transfected with vector encoding US28-NLuc receptor and mVenus-mini-Gα_q (No Nb) and Irr Nb-FLAG, VUN103-FLAG or Nb7-FLAG. Expression of FLAG-tagged intrabodies was induced using 100 nM of tebufenozide, one day prior to read-out. FLAG-tagged intrabody expression was determined by the C-terminal FLAG-tag and an anti-FLAG antibody (n=3 independent experiments). All data is plotted as mean ± S.D. Statistical analyses were performed using unpaired two-tailed t-test. ns, $p > 0.05$. Source data are provided as a Source Data file.

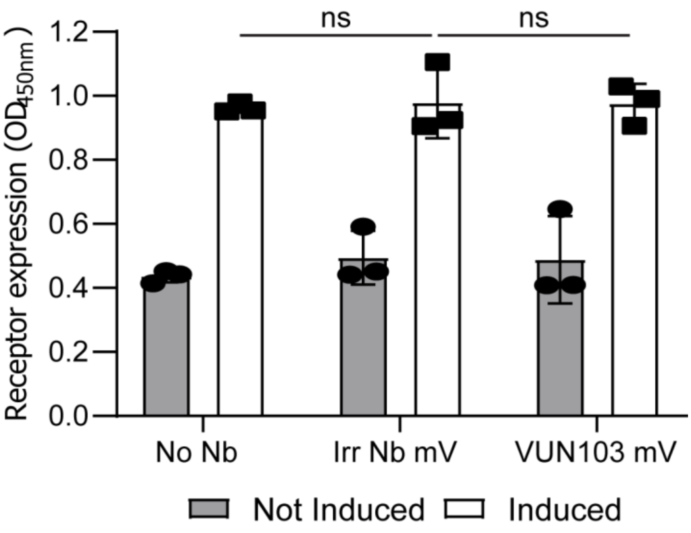

Supplementary Figure 10. Receptor expression of inducible U251 cells. ELISA on U251 cells transduced with doxycycline-inducible US28 (No Nb), doxycycline-inducible US28 and non-US28 targeting intrabody-mVenus (Irr Nb mV) or VUN103-mVenus (VUN103 mV). Receptor expression was not induced or induced with doxycycline and determined by an anti-US28 antibody (n=3 independent experiments). All data is plotted as mean ± S.D. Statistical analyses were performed using unpaired two-tailed t-test. ns, $p > 0.05$. Source data are provided as a Source Data file.

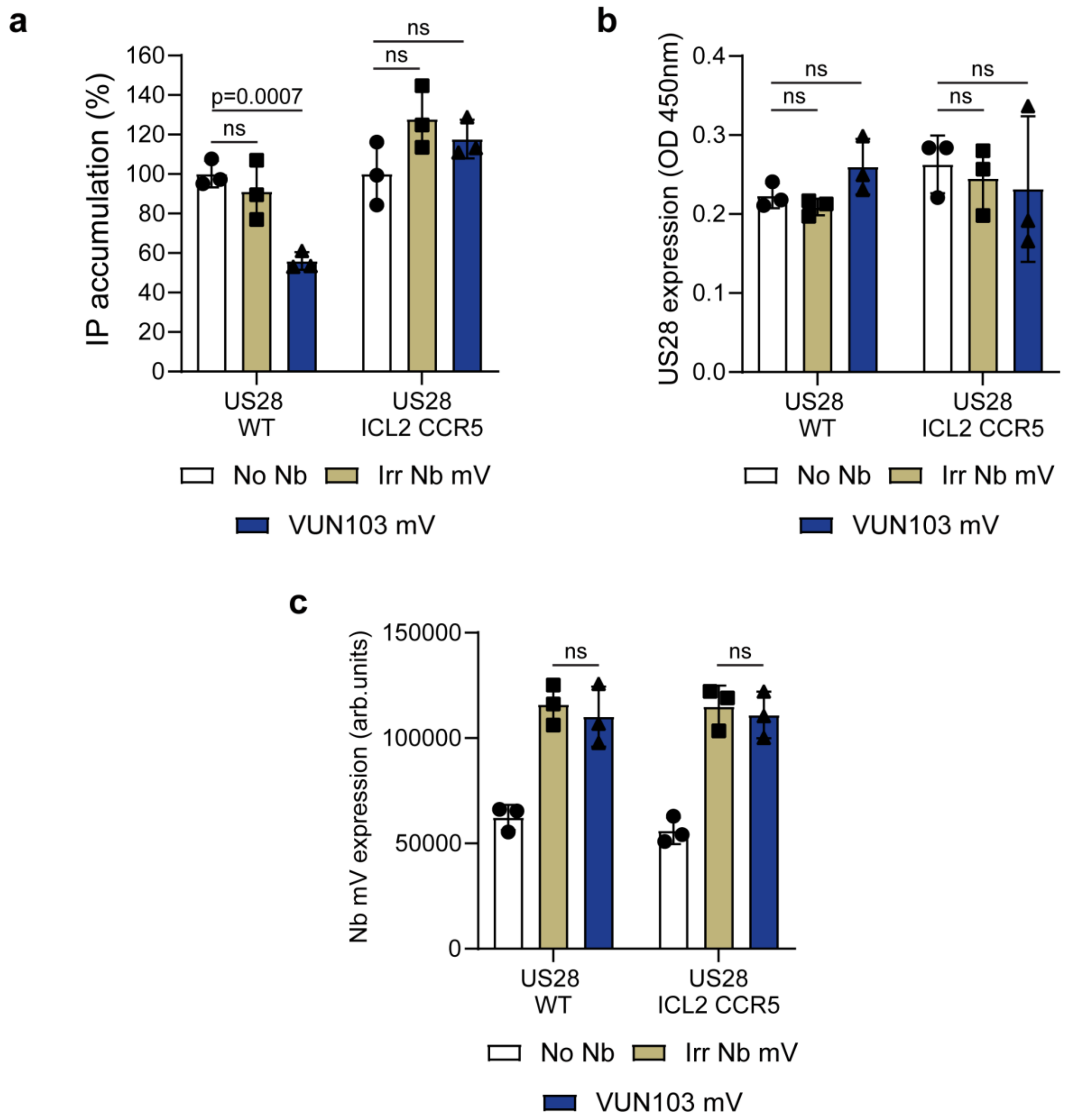

Supplementary Figure 11. Effect of VUN103-mVenus on US28 wildtype and US28 ICL2-CCR5 chimera receptor in U251 cells. a) US28-mediated accumulation of inositol phosphate (IP) in U251 cells transduced with doxycycline-inducible HA-tagged US28 wildtype (US28 WT) and US28 ICL2-CCR5 chimera (US28 ICL2 CCR5) receptor. Cells were either not transfected (No Nb) or transfected with vector encoding a non-US28 targeting intrabody-mVenus (Irr Nb mV) or VUN103-mVenus (VUN103 mV). Inositol phosphate accumulation was normalized to cells expressing US28 WT and no Nb-mVenus construct (n=3 independent experiments). **b)** ELISA on U251 cells transduced with US28 WT and US28 ICL2 CCR5. Cells were not transfected (No Nb) or transfected with vector encoding Irr Nb mV or VUN103 mV. US28 expression was determined using the N-terminal HA-tag and an anti-

HA antibody (n=3 independent experiments). **c)** Determination of the intrabody-mVenus (Nb mV) expression levels of U251 cells transduced with US28 WT and US28 ICL2 CCR5. Cells were not transfected (No Nb) or transfected with vector encoding Irr Nb mV or VUN103 mV. Intrabody-mVenus expression levels were determined by measuring mVenus fluorescence intensity (n=3 independent experiments). All data is plotted as mean ± S.D. Statistical analyses were performed using unpaired two-tailed t-test. ns, $p > 0.05$. Source data are provided as a Source Data file.

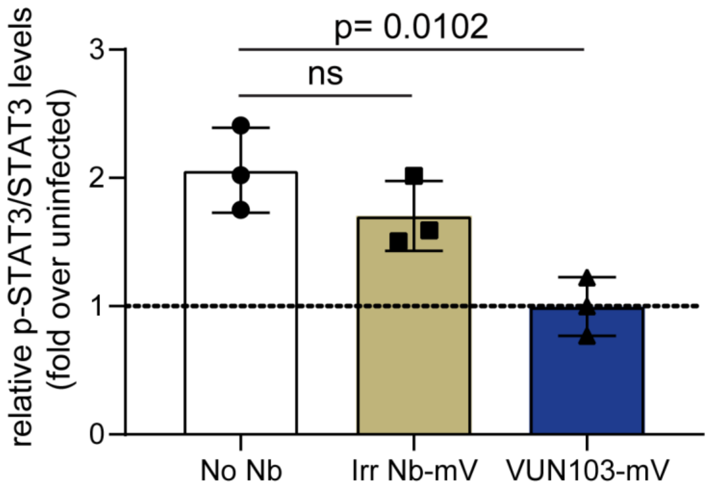

Supplementary Figure 12. Quantification of phospho-STAT3/STAT3 of infected U251 cell lines. Quantification of relative phospho-STAT3 protein levels (p-STAT3) of biological triplicate samples of HCMV-infected U251 cells expressing no nanobody (No Nb), non-US28 targeting-intrabody-mVenus (Irr Nb mV) or VUN103-mVenus (VUN103 mV). Protein levels were normalized to p-STAT3/STAT3levels of uninfected cells. Values were normalized to immediate early/actin levels to normalize for virus infection percentage. Data from 3 independent experiments using independent biological replicates. Data is plotted as mean ± S.D. Statistical analyses were performed using unpaired two-tailed t-test. ns, $p > 0.05$. Source data are provided as a Source Data file.

Supplementary Table 1. Overview of primers used for cloning.

Construct(s)	Primer	Sequence (5' – 3')
pcDEF3-US28-Nb fusions	US28 BamHI fw	5'-CATGATGGATCCGGTGGTGGCGGATCTATGGCAGAGGTGCAGCTG-3'
	Nb XbaI rv	3-ATATTCTAGATTATGAGGAGACGGTGACCTG-5'
pcDNA3.1-NLuc-US28	US28 BamHI fw	5'-ATATGGATCCACACCGACGACGACGAC-3'
	US28 XbaI rv	3'-ATATTCTAGATTACGGTATAATTTGTGAGACG-5'
pcDNA3.1-NLuc-US28-Nb fusions	US28 BamHI fw	5'-ATATGGATCCACACCGACGACGACGAC-3'
	Nb XbaI rv	3'-ATATTCTAGATTATGAGGAGACGGTGACCTG-5'
pEUI(+) Inducible FLAG-tagged intrabodies	Nb EcoRI fw	5'–GATGAATTCGCCACCATGGCAGAGGTGCAG–3'
	Nb FLAG AfIII rv	3'-ATACTTAAGTTATTTGTCGTCGTCGTCTTTGTAGTCCGCTGAGGAGACGGTGACCTG–5'
pLenti6.3 Nanobody-mVenus	Nb BamHI fw	5'–CATGATGGATCCATGGCCGAGGTGCACTTGG–3'
	mVenus XbaI rv	3'-ATCATGTCTAGAGGCTTAGGACTTGTACAGCTCGTC-5'
pcDEF3-Nanobody-mVenus	Nb KpnI fw	5'-CATGATGGTACCGCCACCATGGCAGAGGTGCAGTTGG-3'
	Nb NotI rv	3'-ATCATGGCGGCCGCTGAGGAGACGGTGACCTGG-5'
pcDEF3-Nanobody	Nb KpnI fw	5'-CATGATGGTACCGCCACCATGGCAGAGGTGCAGTTGG-3'
	Nb NotI rv	3'- ATCATGGCGGCCGCTTATGAGGAGACGGTGACCTGG-5'
