# Supplementary material for: Selective targeting of ligand-dependent and -independent signaling by GPCR conformation-specific anti-US28 intrabodies
Source: Nat Commun. 2021 Jul 16;12:4357. doi: 10.1038/s41467-021-24574-y (PMC8285524; doi:10.1038/s41467-021-24574-y)
Supplement: Supplementary file 3 — Reporting Summary [file 41467_2021_24574_MOESM3_ESM.pdf]

natureresearch

Corresponding author(s): Martine J. Smit

Last updated by author(s): Jun 1, 2021

Reporting Summary

Nature Research wishes to improve the reproducibility of the work that we publish. This form provides structure for consistency and transparency in reporting. For further information on Nature Research policies, see Authors & Referees and the Editorial Policy Checklist .

Statistics

For all statistical analyses, confirm that the following items are present in the figure legend, table legend, main text, or Methods section.

n/a	Confirmed	
☐	☒	The exact sample size (n) for each experimental group/condition, given as a discrete number and unit of measurement
☐	☒	A statement on whether measurements were taken from distinct samples or whether the same sample was measured repeatedly
☐	☒	The statistical test(s) used AND whether they are one- or two-sided<br>*Only common tests should be described solely by name; describe more complex techniques in the Methods section.*
☒	☐	A description of all covariates tested
☒	☐	A description of any assumptions or corrections, such as tests of normality and adjustment for multiple comparisons
☐	☒	A full description of the statistical parameters including central tendency (e.g. means) or other basic estimates (e.g. regression coefficient) AND variation (e.g. standard deviation) or associated estimates of uncertainty (e.g. confidence intervals)
☐	☒	For null hypothesis testing, the test statistic (e.g. F, t, r) with confidence intervals, effect sizes, degrees of freedom and P value noted<br>*Give P values as exact values whenever suitable.*
☒	☐	For Bayesian analysis, information on the choice of priors and Markov chain Monte Carlo settings
☒	☐	For hierarchical and complex designs, identification of the appropriate level for tests and full reporting of outcomes
☒	☐	Estimates of effect sizes (e.g. Cohen's d, Pearson's r), indicating how they were calculated

Our web collection on statistics for biologists contains articles on many of the points above.

Software and code

Policy information about availability of computer code

Data collection	•MS/MS spectra were searched against the Swissprot 2019-02 human canonical and isoform FASTA file (42417 entries) using MaxQuant version 1.6.4.0<br>Cox, J., and Mann, M. (2008) MaxQuant enables high peptide identification rates, individualized p.p.b.-range mass accuracies and proteome-wide protein quantification, Nat Biotechnol 26, 1367-1372.
Data analysis	• Data analysis was performed and graphs were plotted with GraphPad Prism version 8.0 for Windows, GraphPad Software, la Jolla California USA, www.graphpad.com<br>• Spheroid assays were analyzed using Fiji version 1.51p for Windows.<br>Schindeling, J.; Arganda-Carrears, I. & Frise, E. et al. (2012), “Fiji: an open-source platform for biological-image analysis”, Nature methods 9(7), 676-682<br>• Confocal images were analyzed using Fiji for Windows and the JACoP Plugin.<br>Bolte, S., and Cordelieres, F. P. (2006) A guided tour into subcellular colocalization analysis in light microscopy, J Microsc 224, 213-232.

For manuscripts utilizing custom algorithms or software that are central to the research but not yet described in published literature, software must be made available to editors/reviewers. We strongly encourage code deposition in a community repository (e.g. GitHub). See the Nature Research guidelines for submitting code & software for further information.

Data

Policy information about availability of data

All manuscripts must include a data availability statement. This statement should provide the following information, where applicable:

- Accession codes, unique identifiers, or web links for publicly available datasets
- A list of figures that have associated raw data
- A description of any restrictions on data availability

Data supporting the findings of this manuscript are available from the corresponding author upon reasonable request. A reporting summary for this study is

available as a Supplementary Information file. All data generated or analyzed during this study are included in this published article. The source Source data are provided as a Source Data file with this paper.

Field-specific reporting

Please select the one below that is the best fit for your research. If you are not sure, read the appropriate sections before making your selection.

☒ Life sciences ☐ Behavioural & social sciences ☐ Ecological, evolutionary & environmental sciences

For a reference copy of the document with all sections, see nature.com/documents/nr-reporting-summary-flat.pdf

Life sciences study design

All studies must disclose on these points even when the disclosure is negative.

Sample size	No sample sizes were calculated beforehand but all experiments were performed with at least 3 technical replicates and each experiment was repeated two to three times. Sample sizes were chosen based on previously published research.
Data exclusions	No data was excluded.
Replication	Each experiment, except for the phage ELISA and confocal microscopy experiment, has been performed three times independently. The phage ELISA screening has been performed one time while the confocal microscopy has been performed twice independently.
Randomization	Randomization of (transfected) cells that were untreated or treated was performed.
Blinding	Acquisition and analysis of data was performed blind.

Reporting for specific materials, systems and methods

We require information from authors about some types of materials, experimental systems and methods used in many studies. Here, indicate whether each material, system or method listed is relevant to your study. If you are not sure if a list item applies to your research, read the appropriate section before selecting a response.

Materials & experimental systems

n/a	Involved in the study	
☐	☒	Antibodies
☐	☒	Eukaryotic cell lines
☒	☐	Palaeontology
☒	☐	Animals and other organisms
☒	☐	Human research participants
☒	☐	Clinical data

Methods

n/a	Involved in the study	
☒	☐	ChIP-seq
☒	☐	Flow cytometry
☒	☐	MRI-based neuroimaging

Antibodies

Antibodies used

• Mouse anti-Myc-tag antibody. Supplier name: Cell Signaling Technology. Catalog number: 2276S. Clone name: 9B11. Lot number: 24. Dilution used: 1:1000
• Rabbit Polyclonal anti-US28 antibody. Supplier name: Custom generated by Covance. Dilution used: 1:1000
• Goat anti-Mouse IgG (H+L)-HRP conjugate antibody. Supplier name: Bio-Rad. Catalog number: 170-6516. Lot number: L005680A. Dilution used: 1:10000
• Goat anti-Rabbit IgG (H+L)-HRP conjugate antibody. Supplier name: Bio-Rad. Catalog number: 170-6515. Lot number: L005679A. Dilution used: 1:10000
• Alexa Fluor® 488- and Alexa Fluor® 546-conjugated Goat anti-Mouse and anti-Rabbit antibodies. Supplier name: Thermo Fisher Scientific. Catalog numbers: A11001, A11030, A11008 and A11010 respectively. Lot numbers: 2090562, 1904466, 1885240 and 1813035. Dilution used: 1:1000
• Rabbit polyclonal anti-pyruvate carboxylase antibody. Supplier: Thermo Fisher Scientific. Catalog Number: PA5-60552. Lot number: 000002248. Dilution used: 1:500
• Rabbit polyclonal anti-Gαq antibody. Provider: Santa Cruz Biotechnology. Catalog number: SC-393. Lot number: 4. Dilution used: 1:1000
• Mouse-anti-actin antibody. Provider: Sigma-Aldrich. Catalog number: A5316. Clone AC-74. Lot number: 059M4770V. Dilution used: 1:2000
• Mouse-Alexa Fluor555 tagged anti-HA antibody. Provider: Thermo Fisher Scientific. Catalog number: 26183-A555. Lot number: TL279653. Dilution used: 1:1000
• Rabbit polyclonal anti-STAT3 antibody. Clone 79D7. Provider: Cell Signaling Technology. Catalog number: 4904. Lot number: 7. Dilution used: 1:1000

• Rabbit polyclonal anti-phospho-STAT3 antibody (Y705). Clone D3A7. Provider: Cell Signaling Technology. Catalog number: 9145. Lot number: 31. Dilution used: 1:1000

• Mouse monoclonal anti-CMV immediate early antigen (IEA). Provider: Millipore. Catalog number: MAB810R. Lot number: 2739234. Dilution used: 1:1000

• Rabbit monoclonal anti-GFP antibody. Provider: Cell Signaling Technology. Catalog number: 2956. Lot number: 4. Dilution used: 1:1000

• Mouse monoclonal anti-FLAG antibody. Provider: Sigma-Aldrich. Catalog number: F1804. Clone M2. Lot number: SLBX2256. Dilution used: 1:1000

• Mouse monoclonal anti-14-3-3 antibody. Provider: Abcam. Catalog number:14108. Clone 3F7. Lot number: GR114413-1. Dilution used: 1:2000

• Rabbit monoclonal anti-beta-arrestin1/2 antibody. Provider: Cell Signaling Technology. Catalog number: 4674. Lot number: 1. Dilution used: 1:1000

• Rabbit anti-Human VEGF antibody (ELISA kit). Provider: Peprotech. Catalog number: 900-T10 Lot number: 0504M010Rb. Dilution used: 1:200

• Biotinylated Rabbit anti-Human VEGF antibody (ELISA kit). Provider: Peprotech. Catalog number: 900-T10BT Lot number: 0504M010Rb. Dilution used: 1:800

Validation

• Mouse anti-Myc-tag antibody. Supplier name: Cell Signaling Technology. Catalog number: 2276S. Clone name: 9B11. Lot number: 24

This antibody was validated for immunoprecipitation and immunofluorescence microscopy by the Cell Signaling Technology. We have validated them by immunofluorescence microscopy on cells transfected with the wild type US28 receptor upon incubation with Myc-tagged nanobodies (Heukers et al, Oncogene, 2018). Cells incubated without Myc-tagged nanobodies served as negative control for specific binding of the anti-Myc-tag antibody (data not shown in manuscript).

• Rabbit polyclonal anti-US28 antibody. Supplier name: Custom generated by Covance.

Method of validation:

This antibody was previously validated by Bongers et al., J Clin Invest, 2010 and Heukers et al, Oncogene, 2018.

• Goat anti-Mouse IgG (H+L)-HRP conjugate antibody. Supplier name: Bio-Rad.

Catalog number: 170-6516. Lot number: L005680A

Method of validation:

Described by: Knapp et al., Elsevier/North Holland Biomedical Press, 1978

• Goat anti-Rabbit IgG (H+L)-HRP conjugate antibody. Supplier name: Bio-Rad.

Catalog number: 170-6515. Lot number: L005679A

Method of validation:

Described by: Knapp et al., Elsevier/North Holland Biomedical Press, 1978

• Alexa Fluor® 488- and Alexa Fluor® 546-conjugated Goat anti-Mouse and anti-Rabbit antibodies. Supplier name: Thermo Fisher Scientific. Catalog numbers: A11001, A11030, A11008 and A11010 respectively. Lot numbers: 2090562, 1904466, 1885240 and 1813035

Method of validation:

We have validated them on immunofluorescence microscopy cells US28 overexpressing cells. Non-expressing served as control.

• Rabbit polyclonal anti-pyruvate carboxylase antibody. Supplier: Thermo Fisher Scientific. Catalog Number: PA5-60552. Lot number: 000002248

Method of validation:

The antibody was tested by Thermo Fisher on immunofluorescence, western blot and Immunohistochemistry. We validated the antibody by pulldown of endogenously biotinylated proteins (including pyruvate carboxylase Ref to add) and subsequent visualization of pyruvate carboxylase using the antibody.

• Rabbit polyclonal anti-Gαq antibody. Provider: Santa Cruz Biotechnology. Catalog number: SC-393. Lot number: 4

Method of validation:

The antibody was previously validated by others including Ngai et al, BMC Immunology, 2009 and Meinychuk et al, Journal of virology, 2004.

• Mouse anti-actin antibody. Provider: Sigma-Aldrich. Catalog number: A5316. Clone AC-74. Lot number: 059M4770V

Method of validation:

The antibody was previously validated by others including Kornblum et al, Nature Genetics, 2013.

• Mouse-Alexa Fluor555 tagged anti-HA antibody. Provider: Thermo Fisher Scientific. Catalog number: 26183-A555. Lot number: TL279653.

Method of validation:

Thermo Scientific validated the antibody by western blot analysis of HA-tagged proteins. We validated the antibody by immunofluorescence microscopy on U251 cells expressing HA-US28 and negative U251 cells.

• Rabbit polyclonal anti-STAT3 antibody. Clone 79D7. Provider: Cell Signaling Technology. Catalog number: 4904. Lot number: 7.

Method of validation:

The antibodies were validated for their use in Western blotting and chromatin immunoprecipitation by Cell Signaling Technology.

• Rabbit polyclonal anti-phospho-STAT3 antibody (Y705). Clone D3A7. Provider: Cell Signaling Technology. Catalog number: 9145. Lot number: 31.

Method of validation:

The antibodies were validated for their use in Western blotting and chromatin immunoprecipitation by Cell Signaling Technology.

• Mouse monoclonal anti-CMV immediate early antigen (IEA). Provider: Millipore. Catalog number: MAB810R. Lot number: 2739234.

Method of validation:

The antibodies were validated for their use in ELISA, Western blotting, immunofluorescence microscopy , immunohistochemistry

and flow cytometry by Merck Millipore. We have validated the antibodies on immunofluorescence microscopy on cells infected with HCMV virus. Non-infected cells served as control.
• Rabbit monoclonal anti-GFP antibody. Provider: Cell Signaling Technology. Catalog number: 2956. Lot number: 4
Method of validation:
The antibodies were validated for their use in Western blotting, immunofluorescence microscopy, immunohistochemistry by Cell Signaling Technology. We have validated the antibodies on western blot using cells transfected with GFP. Non-transfected cells served as control.
• Mouse monoclonal anti-FLAG antibody. Provider: Sigma-Aldrich. Catalog number: F1804. Clone M2. Lot number: SLBX2256
Method of validation:
The antibody was validated for its use in Western blotting, immunofluorescence microscopy, immunoprecipitation, immunohistochemistry by Sigma-Aldrich.
• Mouse monoclonal anti-14-3-3 antibody. Provider: Abcam. Catalog number:14108. Clone 3F7. Lot number: GR114413-1
Method of validation:
The antibody was validated for its use in Immunoprecipitation and Western Blo by Abcam.
• Rabbit monoclonal anti-beta-arrestin1/2 antibody. Provider: Cell Signaling Technologies. Catalog number: 4674. Lot number: 1
Method of validation:
The antibody was validated for its use in Western blotting, immunofluorescence microscopy, immunohistochemistry by Cell Signaling Technology. We have previously validated the antibodies on western blot using cells transfected with beta-arrestin1/2-targeting siRNA, where non-targeting siRNA-transfected cells served as control (Canals et al., PLoS One, 2012).

Eukaryotic cell lines

Policy information about cell lines

Cell line source(s)	HEK293T: derived from fetal human kidney. Obtained from ATCC.<br>U251-MG: derived from a malignant glioblastoma tumor. Obtained from ATCC.
Authentication	The U251-MG cell line was tested for authentication via STR profiling (short tandem repeat analysis) by BaseClear B.V., Leiden, the Netherlands. The results (D7S820: 10,12; CSF1PO: 11,12; THO1: 9.3; D13S317: 10,11; D16S539: 12; vWA: 16,18; TPOX: 8; Amelogenin: X,Y; D5S818: 11,12) correlate with the U251-MG data provided by ATCC and Sigma Aldrich.
Mycoplasma contamination	All cell lines were mycoplasm negative, as determined via PCR by Microbiome (Amsterdam, the Netherlands) and DAPI staining.
Commonly misidentified lines (See ICLAC register)	No commonly misidentified cell lines were used.
